# Supplementary material for: Are carbon emissions trading and green financial instruments synergistic? -Comprehensive quantitative research based on content analysis
Source: PLoS One. 2024 Mar 7;19(3):e0298601. doi: 10.1371/journal.pone.0298601 (PMC10919601; doi:10.1371/journal.pone.0298601)
Supplement: S1 Data — (ZIP) [file pone.0298601.s001.zip › Data/地级市Z_F工作报告.zip!/║┌┴·╜¡/─╡╡ñ╜¡╩╨/└┤╫╘╣1⁄2╓┌║┼í╛┬φ┐╦╩2╛▌═°í┐_2017.docx]

未来五年政府工作总体要求是：高举中国特色社会主义伟大旗帜，全面贯彻党的十八大和十八届三中、四中、五中、六中全会精神，深入贯彻习近平总书记系列重要讲话精神，特别是持续贯彻落实对我省两次重要讲话精神，统筹推进“五位一体”总体布局、协调推进“四个全面”战略布局，牢固树立和贯彻落实新发展理念，全面落实省委省政府战略部署和市十二次党代会工作安排，深入推进改革开放、工业立市、创新驱动，着力打造产业高地、创新新都、开放名市、森林雪乡、幸福家园、清风雪城，如期实现全面建成小康社会目标，奋力开创全面振兴发展新局面。

　　2017年经济社会发展预期目标是：地区生产总值增长7%左右，固定资产投资增长10%以上，规上工业增加值增长7%以上，社会消费品零售总额增长8%以上，城乡居民人均收入均增长7%，一般公共预算收入增长5%，外贸进出口总额增长3%以上，万元GDP综合能耗下降3.5%，经济社会发展整体水平保持全省前列。

　　一、过去五年和2016年工作回顾

　　——综合实力跃上新台阶，在国家和省的地位显著提升。地区生产总值年均增长9.1%，总量跃居全省第三位；人均财力是2011年的1.7倍；绥芬河、海林、穆棱、宁安、东宁跻身全省十强县，林口前移7位。

　　——结构调整取得积极进展，经济发展后劲不断增强。三次产业结构调整为16∶36∶48。我市获“世界黑木耳之都”称号。五年净增规上工业企业82户，工业增加值是2011年的1.6倍，工业税收贡献率达到45%。

　　——深入推进新型城镇化和生态文明建设，城乡面貌发生深刻变化。牡绥快速铁路竣工通车，建成兴隆大桥等桥梁11座、牡海城际等公路16条，综合交通能力进一步提升。五年治理水土流失180万亩，森林覆盖率达到64%，“十大主题公园”、“三溪一河”、江心岛等惠民项目建成使用。

　　——积极融入“龙江丝路带”建设，初步构建起全方位开放新格局。东宁和绥芬河口岸成为宝玉石、粮食和水产品进境指定口岸，全市口岸过货过客能力达到3850万吨、1750万人次，是2011年的3倍和5倍，牡丹江机场出入境旅客突破12万人次。

　　——不断增进民生福祉，人民群众生活水平持续提高。五年民生支出640亿元，是上个五年2.2倍。城镇居民人均可支配收入五年翻一番，农民人均纯收入连续14年领跑全省。

　　——坚定不移深化改革创新，发展环境不断优化。推进大众创业万众创新，五年新增市场主体10.1万户；东宁撤县设市，阳明区享受建制县政策。

　　2016年，我们深入实施“九增行动”，积极推进振兴发展，全市经济稳中有进、积极向好，一些事关全局的工作取得重大突破，实现了“十三五”良好开局。

　　一是产业项目建设取得突破性进展。开复工产业项目1793个、完成投资增长18%，省评价产业项目建设的10项指标7项居全省首位。26个项目列入省“双百工程”，93户企业恢复生产，大工业用电量增长10.8%。

　　二是特色精品农业加快发展。特色农业规模扩张，调减玉米种植面积87.5万亩，新增经济作物41万亩、达到216.7万亩， 新增食用菌2亿袋（块）、达到43.1亿袋（块）， 林下经济、畜牧业产值分别增长6.4%和5.6%。

　　三是现代服务业快速增长。旅游经济增势强劲，推进镜泊湖环湖路、中东铁路运输博物馆等重点项目30个、完成投资28亿元，开工建设养生养老项目19个、总投资26亿元，央视“牡丹江对话法国里昂、对话瑞士日内瓦”等报道进一步提升了城市知名度。

　　四是开放型经济稳步推进。口岸通道更加通畅，牡丹江机场开通国际航空货运，市经开区远东航空监管场所投入运营，绥芬河口岸成为全国首个离境退税陆路口岸。

　　五是城乡基础设施和生态文明建设力度加大。哈牡客专、牡佳客专等重点项目加快建设，虹云桥、太平路贯通、西三条路、牡丹江大桥等标志性工程相继通车。全国文明城市创建和美丽乡村建设加速推进，城市环境整治“八个攻坚战”取得阶段成果。

　　六是人民生活进一步改善。全市民生支出占一般公共预算支出的56%，“九项民生工程”扎实推进，30件利民实事完成年度任务，12个贫困村、1.3万农村贫困人口脱贫。

　　七是政府自身建设得到加强。建立向市委市人大报告、向市政协通报制度，自觉接受监督，办理人大代表议案、建议和政协委员提案504件、办结率100%。

　　二、今后五年主要任务目标

　　未来五年，只要我们抢抓机遇、开拓进取，就一定能在全省新一轮全面振兴发展中继续走在前列。

　　未来五年政府工作总体要求是：高举中国特色社会主义伟大旗帜，全面贯彻党的十八大和十八届三中、四中、五中、六中全会精神，深入贯彻习近平总书记系列重要讲话精神，特别是持续贯彻落实对我省两次重要讲话精神，统筹推进“五位一体”总体布局、协调推进“四个全面”战略布局，牢固树立和贯彻落实新发展理念，全面落实省委省政府战略部署和市十二次党代会工作安排，深入推进改革开放、工业立市、创新驱动，着力打造产业高地、创新新都、开放名市、森林雪乡、幸福家园、清风雪城，如期实现全面建成小康社会目标，奋力开创全面振兴发展新局面。

　　未来五年主要任务和目标是：

　　——经济综合实力显著提升。经济保持中高速发展，全市地区生产总值年均增长7%左右，人均财力、人均收入进入全省第一方阵，主要经济指标增速保持全省前列，县域经济保持全省领先，城区经济快速发展，城市综合竞争力明显提升。

　　——结构调整取得明显进展。加快企业升级改造步伐，深度开发优势资源，大力发展战略性新兴产业，积极培育百亿级产业集群、50亿级龙头企业。加快发展特色高效精品农业，打造全省现代农业“排头兵”。突出发展现代服务业，深入实施“互联网+”行动计划，打造新的经济增长点，服务业增加值占GDP比重达到50%以上。

　　——开放型城市骨架基本成型。深度融入“一带一路”战略和“龙江丝路带”建设，打造东北亚区域合作示范城市。推进铁路、公路、航空重点项目，提升陆海联运能力。坚持出口抓加工、进口抓落地，发展跨境产业集群，培育10家贸易额超亿美元的外向型龙头企业，地产品出口比重、进口资源落地加工率达到20%和60%。

　　——人民生活水平和质量明显提高。全市就业岗位稳步增加，城乡居民收入与经济同步增长，2017年农村贫困人口全部脱贫。完成国家新型城镇化试点建设，实现文明城市、特色城镇、美丽乡村协调发展，城乡居民生活质量不断提升，户籍人口城镇化率提高到65%以上。

　　——生态文明建设取得显著成效。加强生态保护和修复，加大环境综合治理力度，推动资源能源节约利用，水质优良比例达到80%以上、市区空气优良天数达到85%以上、森林覆盖率达到65%以上，万元GDP综合能耗、主要污染物排放总量持续下降。大力发展生态旅游、林下经济、生物医药、清洁能源等绿色产业，打造国际现代山水森林城市。

　　——深化改革取得实质进展。全面推进依法行政，社会治理体系和治理能力现代化取得实质进展，法治政府基本建成。不断优化发展环境，基本建成与百姓和企业互动的“4.0政府”，支持非公经济发展政策机制逐步完善，打造聚人聚财、利于发展的一流环境。

　　三、2017年重点工作任务

　　经济社会发展预期目标是：地区生产总值增长7%左右，固定资产投资增长10%以上，规上工业增加值增长7%以上，社会消费品零售总额增长8%以上，城乡居民人均收入均增长7%，一般公共预算收入增长5%，外贸进出口总额增长3%以上，万元GDP综合能耗下降3.5%，经济社会发展整体水平保持全省前列。

　　（一）抓实产业项目建设，筑牢振兴发展之基。一是创新园区运营模式。推广绥芬河国林木业城经验，加速建设要素集成、一站式服务的产业综合体和主业突出、特色鲜明的专业园区，推进园区规模建设、招商引资、运营管理一体化发展。二是推进产业项目向园区集中。设立产业基金，引导产业项目向市经开区集聚发展。聚焦先进制造业和现代服务业，加强产业招商和项目建设，年内引进亿元以上项目30个，开复工亿元以上项目74个。三是加大精准招商力度。全市引进亿元以上项目100个、10亿元以上项目10个，引进域外资金增长10%。四是加快重点项目建设。完善领导包保、定期会办等工作机制，加速推进立市型、税源型项目，全市新开工10亿元以上工业项目5个以上。

　　（二）做好“三篇大文章”，推动工业转型升级。一是把“三篇大文章”落实到产业和项目。改造提升“老字号”，积极申报国家级产业转型示范区。深度开发“原字号”，围绕优势资源新上一批精深加工项目。培育壮大“新字号”，积极推进太敬机器人等重点项目，引进战略投资者深度开发石墨资源。二是深入推进大众创业万众创新。深入落实省“千户科技型企业三年行动计划”，新增销售收入500万元以上科技型企业100户以上。建立“牡丹江孵化器创新服务联盟”，新增科技企业孵化器和众创空间各10家以上。全市高新技术产业增加值增长10%以上。三是壮大民营经济。深入落实省“非公经济发展三年行动计划”和“中小微企业成长工程”，制定出台支持民营经济和中小微企业发展政策措施，降低企业制度性交易成本，全市非公经济贡献率达到64.5%，新增小微企业1000户以上。

　　（三）壮大特色高效精品农业，争当全省现代农业排头兵。一是推进绿特产品规模扩张，以市场为导向调好调顺调优农业结构。重点建设一批“菜菌药果烟”大型基地和特色旅游村镇，全市调减玉米50万亩以上，新增经济作物30万亩，食用菌栽培规模保持43亿袋（块），蔬菜面积、林下经济和畜牧业产值分别增长30%、7%和5%以上，农业旅游收入实现翻番、达到15亿元。二是从加工、质量、品牌、营销入手，提升农业产业化和竞争力。完善农产品质量监管体系，5个县（市）农产品检测中心投入使用，完成“三减两增一提升”万亩示范田35个。 构建线上线下并重、优质优价结合的农业市场营销体系， 全市农产品线上销售增长35%以上。 三是强化农业服务、深化农业改革，促进农业适度规模经营。全面完成土地承包经营权确权登记，探索农村承包土地“三权分置”办法。发展壮大各类新型经营主体，家庭农场、农民专业合作社分别达到1260个和2250个。

　　（四）加快发展现代服务业，进一步释放消费需求潜力。一是大力发展旅游业。编制牡丹江全域旅游规划和冰雪文化旅游专项规划，修编镜泊湖总体规划，全力打造镜泊湖、中国雪乡两大品牌，建设北国风光生态旅游胜地。二是加快商贸业转型升级。创新商业业态模式，辟建大宗商品现货交易市场。高标准编制商业网点规划。推进市经开区国家电子商务示范基地、中国食品俄罗斯海外仓等项目，开工建设单体投资30亿元以上文化旅游综合体项目。三是着力发展物流业。按照打造国际物流集散枢纽的目标，科学规划专业性物流园区。加快推进牡丹江机场货运监管场所、华晟物流中心项目二期、俄罗斯水产品批发市场等项目建设。四是积极发展金融、文化、健康、房地产等产业。实施“中西名医引进计划”，打造心血管病、医学美容、中医诊疗等特色专科，培育发展大健康产业。加强房地产市场监管，推进房地产业平稳健康发展。

　　（五）大力发展开放型经济，构建以俄韩为重点的大开放格局。一是进一步畅通经贸通道。大力推进铁路、公路、机场项目，加快哈牡客专、牡佳客专、哈牡既有线电气化改造项目建设，加速谋划推进牡敦客专项目，完成牡符铁路预可研编制工作；开工建设东珲高等级公路。二是进一步壮大外向型产业。依托东宁、绥芬河进境食用水生动物指定口岸，建设全省首个俄海产品加工基地。三是进一步做强开放平台。牡丹江保税物流中心通过验收并投入使用，牡丹江综合保税区力争获批。穆棱边境经济合作区、中韩国际合作示范区建设取得实质进展。四是进一步扩大交流合作。建设俄韩多语标识和语音服务平台。办好牡丹江中俄国际啤酒文化节、东宁宝玉石文化节。

　　（六）推进城乡和生态文明建设，打造宜居宜业美丽家园。一是以创建全国文明城市为主线，加快中心城市建设。加强市政建设，实施市区13个铁路道口平改立工程，完成西十一条路跨铁立交桥和西环江路建设，整修16条背街巷路、让百姓出行更加便捷，力争新火车站南站房建成，提升市区沿江两岸亮化水平。二是以国家新型城镇化试点为引领，推进小城镇和美丽乡村建设。发挥16个国家重点镇及“响水两化”示范引领作用，城关镇、重点镇完成基础设施投资9亿元、完成棚户区改造30万平方米。加快推动美丽乡村建设，全市建设美丽乡村示范村20个、农村示范社区20个。三是以国家生态文明先行示范区为载体，加强生态文明建设。深入开展空气、水体、土壤、固废、辐射、噪音、污染综合治理，全面落实“河长制”，流域水质和市区空气优良天数好于同期，主要污染物总量减排超额完成省定目标。

　　（七）着力保障和改善民生，不断提高群众幸福指数。一是更有力更扎实推进脱贫攻坚工作。严格落实“六个精准”和“五个一批”要求，构建短期能脱贫、长期能致富的扶贫攻坚模式，全市22个村、2.45万农村人口到2017年底全部脱贫。二是抓好棚户区改造和保障性住房建设。推进铁岭等棚户区改造项目，改造棚户区8465户、安置回迁居民6000户。三是强化就业增收和社会保障。深入实施就业优先战略，抓好重点人群就业，鼓励城乡居民通过创新创业增收致富，新增城镇就业5.4万人，城镇登记失业率控制在4.3%以内。四是大力发展社会事业。实施“健康牡丹江”行动，构建现代医疗卫生体系和食品药品监管体系，医联体县域内就诊率达到80%以上，基层卫生机构与养老机构签约率达到100%。五是加强和创新社会治理。开展“抽检监测规范年”活动，争创国家级食品安全城市。深入推进“平安牡丹江”建设，健全社会治安立体化防控体系，增强防范处置各类突发事件能力，严防严打各类刑事犯罪，争创“全国平安市”。

　　（八）推进政府改革创新，提升施政能力和服务水平。一是深化行政审批制度改革和政府机构改革，促进科学、高效理政。以打造“4.0政府”为方向，持续深化行政审批制度改革，组建行政审批局，推动政府服务流程再造，推行“五证合一”企业登记模式，实现“一屏妥”手机移动审批服务。二是加快法治政府建设，促进依法、规范行政。严格执行政府重大决策法定程序，推进行政决策科学化、民主化、法治化。全面推进政务公开，扎实推进电子政务建设，促进政府施政更加透明高效。三是加强政府自身建设，促进从严、廉洁治政。严格执行中央关于党内政治生活、廉洁自律、党内监督、纪律处分等“准则”和“条例”，巩固拓展“两学一做”学习教育成果，不断开创政府系统党组织全面从严治党新局面。
